# Supplementary material for: Resolving the full spectrum of human genome variation using Linked-Reads
Source: Genome Res. 2019 Apr;29(4):635–45. doi: 10.1101/gr.234443.118 (PMC6442396; doi:10.1101/gr.234443.118)
Supplement: Supplemental Material [file supp_gr.234443.118_Supplemental_longranger-2.2.zip › longranger-2.2.tar.bz2!/lr2.2-public/lib/go/src/code.google.com/vitess/vitess.io/_layouts/archive.html]

---
layout: default
---

{% if page.image.feature %}

{% if page.image.credit %}{% include image-credit.html %}{% endif %}

{% endif %}

# {{ page.title }}

{% if page.excerpt %}

## {{ page.excerpt }}

{% endif %}

{{ content }}
